# Endolysosomal TPCs regulate social behavior by controlling oxytocin secretion

**DOI:** 10.1073/pnas.2213682120

**Published:** 2023-02-06

**Authors:** Lora L. Martucci, Jean-Marie Launay, Natsuko Kawakami, Cécile Sicard, Nathalie Desvignes, Mbarka Dakouane-Giudicelli, Barbara Spix, Maude Têtu, Franck-Olivier Gilmaire, Sloane Paulcan, Jacques Callebert, Cyrille Vaillend, Franz Bracher, Christian Grimm, Philippe Fossier, Sabine de la Porte, Hirotaka Sakamoto, John Morris, Antony Galione, Sylvie Granon, José-Manuel Cancela

**Affiliations:** ^a^Neuroscience Paris-Saclay Institute, CNRS UMR 9197, Paris-Sud University, Paris-Saclay University, Saclay 91400, France; ^b^Université Paris-Saclay, Université de Versailles Saint-Quentin-en-Yvelines, Inserm, Evolution of Neuromuscular Diseases: Innovative Concepts and Practices, Versailles 78000, France; ^c^Department of Pharmacology, University of Oxford, Oxford OX1 3QT, UK; ^d^INSERM UMR-S 942, Hôpital Lariboisière, Paris Cedex 10 75475, France; ^e^Ushimado Marine Institute, Graduate School of Natural Science and Technology, Okayama University, Ushimado, Setouchi, Okayama 701-4303, Japan; ^f^Walther Straub Institute of Pharmacology and Toxicology, Faculty of Medicine Ludwig-Maximilians-University, Munich 80336, Germany; ^g^Laboratoire de Biochimie et Biologie Moléculaire, Hôpital Lariboisière, Paris 75010, France; ^h^Inserm UMR-S 1144 Universités Paris Descartes-Paris Diderot, Optimisation Thérapeutique en Neuropsychopharmacologie - Faculté des Sciences Pharmaceutiques et Biologiques, Paris Descartes, Paris Paris 75006, France; ^i^Department of Pharmacy - Center for Drug Research, Ludwig-Maximilians University, Munich 81377, Germany; ^j^Department of Physiology, Anatomy & Genetics, University of Oxford, Oxford OX1 3QX, UK

**Keywords:** calcium, channel, NAADP, hypothalamus, neuropeptide

## Abstract

Oxytocin (OT) is a prominent regulator of many aspects of mammalian social behavior and stored in large dense-cored vesicles (LDCVs) in hypothalamic neurons. It is released in response to activity-dependent Ca^2+^ influx, but is also dependent on Ca^2+^ release from intracellular stores, which primes LDCVs for exocytosis. Despite its importance, critical aspects of the Ca^2+^-dependent mechanisms of its secretion remain to be identified. Here we show that lysosomes surround dendritic LDCVs, and that the direct activation of endolysosomal two-pore channels (TPCs) provides the critical Ca^2+^ signals to prime OT release by increasing the releasable LDCV pool without directly stimulating exocytosis. We observed a dramatic reduction in plasma OT levels in TPC knockout mice, and impaired secretion of OT from the hypothalamus demonstrating the importance of priming of neuropeptide vesicles for activity-dependent release. Furthermore, we show that activation of type 1 metabotropic glutamate receptors sustains somatodendritic OT release by recruiting TPCs. The priming effect could be mimicked by a direct application of nicotinic acid adenine dinucleotide phosphate, the endogenous messenger regulating TPCs, or a selective TPC2 agonist, TPC2-A1-N, or blocked by the antagonist Ned-19. Mice lacking TPCs exhibit impaired maternal and social behavior, which is restored by direct OT administration. This study demonstrates an unexpected role for lysosomes and TPCs in controlling neuropeptide secretion, and in regulating social behavior.

## Significance

Oxytocin vesicles are released from the neurohypophysis into the bloodstream to regulate reproductive physiology and from the hypothalamus to control social behavior. The number of oxytocin vesicles exocytosed during these physiological processes are greatly potentiated by priming whose mechanism is not well understood. Here we find that endolysosomal two-pore channels (TPCs) are an integral component of the priming process so that in the absence of TPCs, plasma oxytocin levels and hypothalamic release fall to very low levels. TPC deletion also leads to social defects and suggests that TPC dysfunction might contribute to social behavioral disorders. Finally, since hormone replacement therapies are largely ineffective, pharmacological TPC-driven Ca^2+^ release may provide a promising strategy for boosting oxytocin signaling in social disorders.

Oxytocin (OT) was initially described as a hormone regulating parturition (1, 2) and lactation (3), but it is now also well-recognized as a prosocial hormone regulating maternal behavior (4, 5), social recognition (6–8), and social interactions (9–14). OT is stored in large dense-core vesicles (LDCV) in the magno- and parvocellular secretory neurons of the paraventricular nuclei (PVN) and the supraoptic nuclei (SON) of the hypothalamus. This neuropeptide is released from hypothalamic neurons into different brain structures, and via the pituitary gland into the systemic bloodstream. OT neurons are multifunctional multisensory cells that respond to a wide variety of stimuli to fulfil their numerous roles described above (for review see ref. 15). However, the regulatory mechanisms underlying OT secretion are still unclear. Although OT secretion is known to be independently regulated in different neuronal compartments such as axons, soma, and dendrites (8, 16–20), the intracellular signaling pathways underlying secretion in these complex multitask neurons are still unclear.

OT release from both somatodendritic sites (16) and neurohypophysial axonal terminals (21) have been proposed to be dependent on Ca^2+^ release from intracellular stores in addition to Ca^2+^ influx via voltage-dependent Ca^2+^ channels. Indeed release of OT from pituitary axonal terminals and hypothalamus was shown to be highly dependent on the expression of cluster of differentiation 38 (CD38) (21, 22), a transmembrane multifunctional enzyme that catalyzes the synthesis of the Ca^2+^ mobilizing messenger, cyclic ADP-ribose (cADPR). cADPR which promotes Ca^2+^ release via ryanodine receptors (RyRs) from the endoplasmic reticulum (ER) (23) caused a large increase in OT release from isolated oxytocinergic nerve endings (21). However, CD38 also may catalyze the synthesis of another major Ca^2+^ mobilizing messenger, nicotinic acid adenine dinucleotide phosphate (NAADP), which specifically mobilizes Ca^2+^ from acidic organelles such as lysosomes (24, 25) via two-pore channels (TPCs) (26). However, in contrast to cADPR, NAADP was found not to directly stimulate OT release (21).

Somatodendritic OT secretion has also been shown to exhibit the important property of priming, which greatly increases the extent of exocytosis of OT-containing LCDVs (16). Priming in neuroendocrine cells is the phenomenon whereby an initial signal prepares cells for an anticipated subsequent trigger, sometimes involving the autocrine action of the peptide released (8, 16, 27). It is of fundamental significance in the neuroendocrine system where such a mechanism mediates such phenomena as the OT-controlled milk-ejection reflex and the LH surge at ovulation (28). In hypothalamic neurons, this initial priming signal can be OT itself (self-priming) or other hormones or neurotransmitters which activate metabotropic cell surface receptors to mobilize Ca^2+^ from intracellular stores. This results in a substantial augmentation of the secretory response to subsequent cell activation and is thought to occur by recruiting a reserve pool of LCDVs to the plasma membrane, increasing their probability of release. It had been previously suggested that Ca^2+^ release from Ca^2+^ storage organelles primes vesicles for sustained OT release since pharmacological release of Ca^2+^ from the ER by the sarcoendoplasmic reticulum Ca^2+^ ATPase (SERCA) inhibitor thapsigargin and consequential Ca^2+^ entry promoted priming effects (16, 29, 30).

Lysosomes are recognized as important organelles in autophagic macromolecular degradation and membrane repair, but they also contain a high concentration of Ca^2+^ that could be mobilized for cell signaling (24, 25, 31, 32). Electron microscopy has shown the presence of lysosomes in axon endings, dendrites, cell-bodies, and Herring bodies of OT neurons located in areas rich in LDCVs (33–35), but so far, their role in hypothalamic neurons as a source of Ca^2+^ has remained unexplored. In various cell types, endosomes and lysosomes release Ca^2+^ through the activation of the endolysosomal TPCs, modulated by NAADP (26, 36–39) and phosphatidylinositol-3, 5-bisphosphate (PI(3, 5)P_2_) (40, 41). TPCs belong to an ancient cation channel family present in numerous species with three known isoforms (TPC1, TPC2, and TPC3). In humans and rodents, only TPC1 and TPC2 are expressed, displaying different ionic selectivity in part depending on how they are activated: NAADP favoring Ca^2+^ permeation (42, 43), while PI(3, 5)P_2_ favors Na^+^ permeation (40, 43–45). TPCs have been implicated in many aspects of cell signaling, autophagy, and vesicular trafficking in various cell types (42, 46, 47), but despite the growing interest in these channels, their roles in the central nervous system remain largely unexplored (for review, see ref. 22).

Here on the basis that CD38 is a major regulator of OT secretion (21), we have examined the role of endolysosomal TPCs as the principal targets of the NAADP branch of the CD38 signaling pathway and found that they play critical roles in the regulation of OT secretion and thus control social behavior.

## Results

### TPCs Are Expressed in the Hypophysis and Hypothalamus and Their Deletion Leads to a Dramatic Reduction in Plasma OT Levels.

**Fig. 1.**
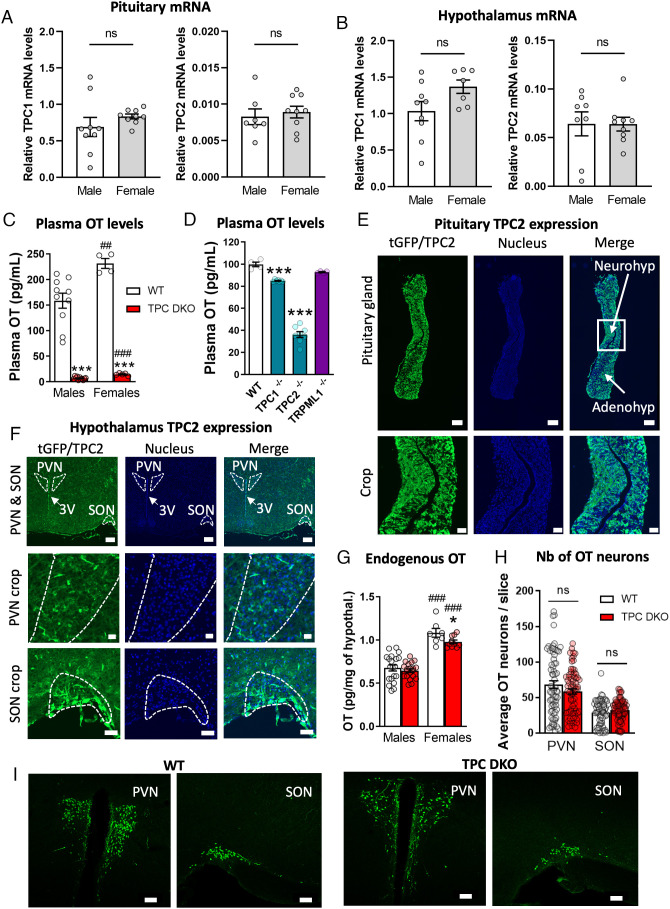
Plasma OT levels are altered in TPC knockout mice. (*A*) qPCR analysis in duplicate of TPC1 and TPC2 mRNA in the hypophysis of WT mice (each group n = 9 mice). (*B*) qPCR analysis in duplicate of TPC1 and TPC2 mRNA in the hypothalamus of WT mice (each group n = 9 mice). (*C*) Plasma OT levels (male WT n = 10, male DKO n = 11, female WT n = 4, female DKO n = 5 mice). *Refers to differences between TPC DKO mice and their respective WT. ^#^Refers to differences between females and their respective strain males. (*D*) Plasma levels of OT in *Tpcn1^−/−^ (*TPC1^−/−^), *Tpcn2^−/−^* (TPC2^−/−^) and mucolipin1 (*Trpml1^−/−^*) KO mice (WT n = 4; TPC1^−/−^ n = 8; TPC2^−/−^ n = 8; TRPML1^−/−^ n = 4 mice). (*E*) Immunostaining of TPC2-tGFP reporter (in green) revealed with the anti-GFP antibody and nuclei stained with bisbenzimide (in blue) in the hypophysis containing the neurohypophysis (neurohyp) and adenohypophysis (adenohyp) (n = 2 mice). (*Top* scale bar, 200 µm.) *Lower* panel represents a magnification of the *Top* panel images (white square), (scale bar, 50 µm.) (*F*) Immunostaining of TPC2-tGFP reporter (in green) and nuclei stained with bisbenzimide (in blue) in the hypothalamus. Top raw shows the PVN, SON, and third ventricle (3V). The middle row shows a crop of the PVN, and the lower row shows a crop of the SON (n = 2 mice). (*Top Row* scale bar, 200 µm.) (*Middle row* scale bar, 20 µm.) (*Bottom Row* scale bar, 50 µm.) (*G*) Endogenous OT content in isolated hypothalami (male WT n = 20, male DKO n = 20, female WT n = 7, female DKO n = 10 mice). (*H*) Average number of OT neurons per slice in the PVN and SON in male mice (each group n = 7 mice) and illustrated in *I*. (*I*) Immunostaining of OT neurons from the PVN of SON of WT and TPC DKO mice. Statistical analysis were performed with unpaired *t* test (*A*–*C*, *G* and *H*) and one-way ANOVA followed by the post hoc Dunnett’s multiple comparison test (*D*). Values are expressed as mean ± SEM. *P < 0,05; **P < 0.01; ***P < 0.001 and ns, nonsignificant.

We first performed RT-qPCR both in male and female mice to quantify the mRNA levels of TPC1 and TPC2 gene transcripts in the pituitary gland and in the hypothalamus that contain the oxytocinergic neurons projecting to the pituitary. The data indicate that both TPC1 and TPC2 are equally expressed in the pituitary gland (Fig. 1*A*) and the hypothalamus (Fig. 1*B*) in male and female mice. OT being a major neuropeptide released from the pituitary gland, we explored if TPCs could play a role in OT signaling by generating mice lacking both TPC1 and TPC2 [TPC double knockouts (DKO)], the two isoforms expressed in mice. These mice were viable and grew normally (48). In both wild-type (WT) and TPC DKO mice, we then measured plasma OT level as an indicator of pituitary secretory activity. Strikingly, we found a dramatic decrease of around 95% of OT plasma concentrations in both male and female TPC DKO mice when compared with WT mice (Fig. 1*C*). As expected, WT female mice showed higher plasma OT levels than WT male mice and the same was true for TPC DKO mice, although absolute levels were substantially reduced in TPC DKO mice. The decrease in plasma OT is specific to TPCs since mice lacking TRPML1 (TRPML1^−/−^ mice), another lysosomal cation channel, showed no significant reduction in OT plasma levels (Fig. 1*D*). Experiments with single TPC KO mice i.e., TPC1^−/−^ and TPC2^−/−^ indicated that both isoforms of TPC regulate the OT plasma levels with a more prominent role for TPC2 (Fig. 1*D*). This is supported by the high level of TPC2-GFP staining observed in the neurohypophysis (Fig. 1*E* and *SI Appendix*, Fig. S1) and hypothalamus (Fig. 1*F* and *SI Appendix*, Fig. S1) of a mouse (49) expressing the gene reporter green fluorescent protein (GFP) along with TPC2 gene (tGFP-TPC2 mice). Since in both TPC1^−/−^ and TPC2^−/−^ mice, the plasma OT levels were significantly reduced, we decided to focus on TPC DKO mice to investigate the role of TPCs as an ion channel family in OT secretion.

The reduction in OT plasma levels observed in TPC DKO mice (Fig. 1 *C* and *D*) could be due to either a deficit in OT synthesis or an altered OT secretion. Therefore, we first tested whether the synthesis of OT was impaired by measuring the endogenous OT content of the ex vivo isolated hypothalami. We found no difference in the OT content in TPC DKO males and a modest reduction in OT content in TPC DKO females, compared with WT mice (Fig. 1*G*). Furthermore, similar numbers of OT neurons were present in the PVN and the SON in the two strains (Fig. 1*H*, illustrated in Fig. 1*I*). These results clearly indicated that TPC deletion has little impact on OT synthesis or content of brain nuclei expressing the neuropeptide.

### Deletion or Pharmacological Inhibition of TPCs Leads to OT Secretion Defects from the Hypothalamus.

**Fig. 2.**
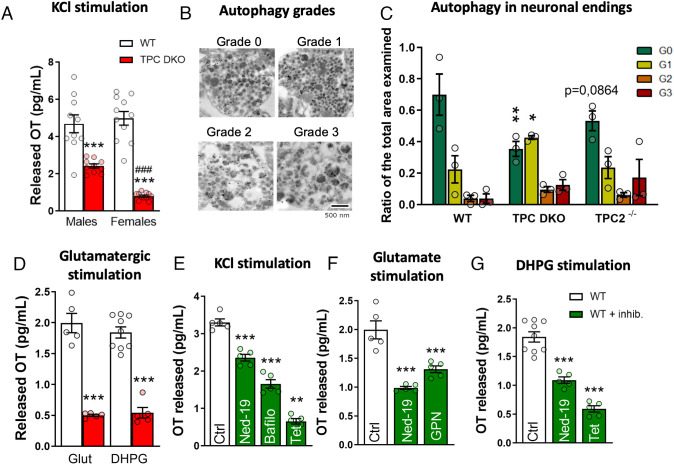
Pharmacological inhibition (green) or TPC deletion (red) reduced OT release. (*A*) OT released from isolated hypothalami after 50 mM KCl stimulation (each bar group n = 5 mice). *Refers to differences between TPC DKO mice and their respective WT. ^#^Refers to differences between males and females of the same mouse strain. (*B*) Electron microscopy images labeled with neurophysin-I (15-nm gold particles) to distinguish OT-positive endings depicting autophagic grades: grade 0 corresponds to an absence of autophagy vacuoles within the ending, whereas grades 1, 2, and 3 correspond to a progressive increase of autophagic vacuoles. (*C*) Histograms of the electron microscopy showing the ratio of the area occupied by different grades of autophagic bodies in the neurohypophysial nerve endings divided by the total area of the ending (each group n = 3). (*D*) OT released from isolated hypothalami after 1 mM glutamate stimulation (male WT n = 20, male DKO n = 20) and after mGluR1 stimulation with 100 µM DHPG (each bar group n = 5 mice). (*E*) OT released from isolated hypothalami after 50 mM KCl stimulation in presence of 100 µM Ned-19, 4 µM bafilomycin A1, or 10 µM tetrandrine (each group n = 5 mice). (*F*) OT released from isolated hypothalami after 1mM glutamate stimulation in presence of 100 µM Ned-19 or 50 µM GPN (each bar group n = 5 mice). (*G*) OT released from isolated hypothalami after 100 µM DHPG (mGluR1) stimulation in presence of 100 µM Ned-19 or 10 µM tetrandrine (each bar group n = 5 mice). Statistical analyses were performed with an unpaired *t* test (*A* and *D*); ANOVA 2-way (*C*) or ANOVA 1-way (*E*–*G*) followed by post hoc Dunnett’s test. Values are expressed as mean ± SEM ns (no significant); *P < 0.05; **P < 0.01; ***P < 0.001 or ^###^*P* < 0.001 (difference between female and male).

To explain the large decreases in plasma OT levels, we then examined whether OT release was impaired. We found that in the absence of stimulation, the basal release from isolated hypothalami was reduced in TPC DKO (*SI Appendix*, Fig. S2). Furthermore, we stimulated hypothalami from male and female TPC DKO mice with high potassium chloride (KCl) concentrations to depolarize neurons and promote somatodendritic exocytosis of neuropeptide vesicles and found that OT release was reduced by around 50% for male TPC DKO and by 84% for females, compared with WT (Fig. 2*A*). Because of the fall in plasma OT levels (Fig. 1*C*) and the dramatically impaired hypothalamic OT release in TPC DKO mice (Fig. 2*A*), we performed electron microscopy of the neurohypophysis to examine the presence and morphology of secretory vesicles at the nerve endings in TPC DKO mice. Since nonreleased vesicles age and undergo autophagic changes, we quantified the morphology and autophagy of these aged vesicles in neurohypophysis nerve endings as an index of nonreleased vesicles. We scored the autophagic vesicle status as follows: grade 0 corresponded to an absence of autophagic vacuoles within the ending, whereas grades 1, 2, and 3 corresponded to a progressive increase in autophagic vacuoles as a consequence of an increased number of aged OT vesicles (Fig. 2*B*). In agreement with the dramatically low plasma OT levels, neuron endings in TPC DKO contained more autophagic vesicles (grade 1, 2, and 3) as compared with WT neurons (Fig. 2*C*). Of note, in line with the less marked drop in plasma OT levels from TPC2^−/−^ mice (Fig. 1*D*), the autophagic phenotype was less severe in TPC2^−/−^ mice compared with TPC DKO mice supporting a role for TPC1 (Fig. 1*C*).

Next, we examined glutamate-evoked secretion of OT, since physiologically, OT neurons receive a synaptic glutamatergic input to trigger neuropeptide secretion (50). We focused on type 1 metabotropic glutamate receptors (mGluR1) stimulation as it is known to trigger OT secretion (50). In our experiments on isolated hypothalami, both glutamate and (S)-3,5-Dihydroxyphenylglycine (DHPG), a selective agonist of mGluR1 that has previously been proposed to recruit the NAADP–TPC pathway (31), elicited OT release to a similar degree in WT mice (Fig. 2*D*). Under the same conditions, the OT release evoked by DHPG was greatly reduced by around 75% in TPC DKO mice (Fig. 2*D*). To test whether the exocytic mechanisms were themselves impaired, we used the Ca^2+^ ionophore ionomycin to generate a large cytosolic Ca^2+^ increase, bypassing the physiological mechanisms to trigger secretion. Ionomycin elicited OT secretion in a comparable extent in both WT and TPC DKO hypothalami, showing no impairment in the OT exocytosis machinery per se (*SI Appendix*, Fig. S3). Our results indicate that the deletion of TPCs substantially prevented the release of OT, rather than disrupting OT synthesis or packaging in vesicles.

Next, we examined the effects of pharmacological inhibition of the TPC pathway on both KCl- and agonist-evoked neuropeptide release from isolated hypothalami from WT mice to see if the effects in the TPC DKO phenocopied the effects of TPC inhibitors. We antagonized NAADP-activation of TPCs either with Ned-19, a membrane-permeant selective NAADP inhibitor (51), or by blocking TPC directly with tetrandrine, a Ca^2+^ channel blocker found to be a potent TPC inhibitor (52). We also disrupted the lysosomal Ca^2+^ storage with bafilomycin A1 (H^+^-V-ATPase inhibitor) or permeabilized the lysosomal membrane with GPN, a lysosomotropic agent (24, 37, 53). Fig. 2*E* shows that, upon KCl stimulation, OT release was significantly reduced by Ned-19 (−30%), bafilomycin A1 (−50%), and tetrandrine (−80%). We also examined agonist-evoked OT secretion and observed a significant reduction in OT release evoked by glutamate in the presence of Ned-19 (−55%) and GPN (−30%) (Fig. 2*F*). Upon DHPG stimulation, we also found a significant decrease in OT release of more than 40% in the presence of Ned-19, and a reduction of almost 70% in the presence of tetrandrine, compared with their respective controls (Fig. 2*G*). These results provide strong evidence that TPCs are required for OT release and that glutamate-induced secretion likely depends on mGluR1.

### Pharmacological Inhibition of the TPC-Pathway Shows That Glutamate-Evoked Ca^2+^ Responses Are TPC-Dependent.

**Fig. 3.**
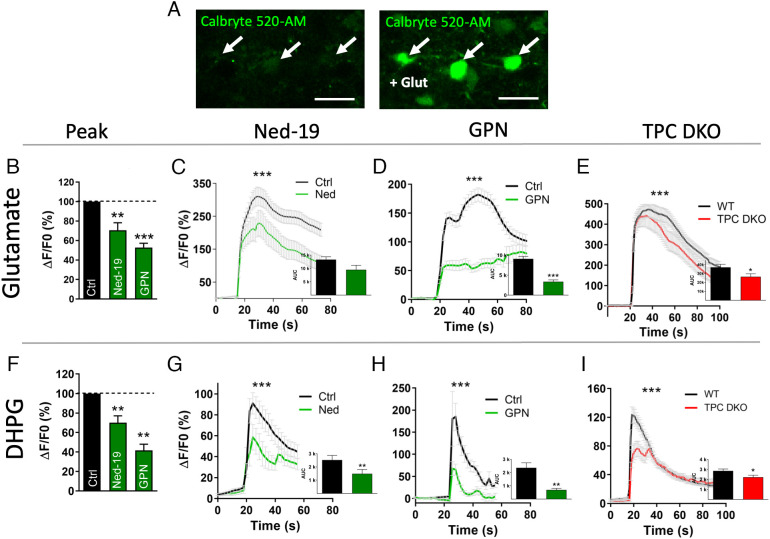
Pharmacological inhibition (green) or deletion of TPCs (red) reduced agonist-evoked Ca^2+^ response in the PVN of the hypothalamus. (*A*) Example of confocal Ca^2+^ imaging of WT neurons before and after glutamate stimulation. Acute slices were incubated with the Ca^2+^ sensitive probe Calbryte 520-AM. Arrows show some examples of neurons responding to 1 mM glutamate stimulation. (Scale bar, 50 µm.) (*B*) Shows the peak amplitude and (*C* and *D*) shows the time course graphs of glutamate-induced Ca^2+^ response in presence of 100 µM Ned-19 (green line, Ctrl n = 20, Ned-19 n = 20 cells, both n = 3 mice) or 50 µM GPN (green line, Ctrl n = 123, GPN n = 123 cells, both n = 4 mice) in hypothalamic neurons of WT mice compared to untreated control WT hypothalamic neurons (Ctrl, black line),  area under the curve (AUC). (*E*) Glutamate-induced Ca^2+^ response in hypothalamic neurons of TPC DKO mice (red line, n = 47 cells, n = 5 mice) compared to control WT hypothalamic neurons (Ctrl, black line, n = 67 cells, n = 5 mice),  area under the curve (AUC). (*F*) Shows the peak amplitude and (*G* and *H*) show the time course graphs of DHPG-induced Ca^2+^ response in the presence of 100 µM Ned-19 (green line, Ctrl n = 19, Ned-19 n = 19 cells, both n = 7 mice) or 50 µM GPN (green line, Ctrl n = 8, GPN n = 8, both n = 5 mice) in hypothalamic neurons of WT mice compared with untreated control WT hypothalamic neurons (Ctrl, black line),  area under the curve (AUC). (*I*) DHPG-induced Ca^2+^ response in hypothalamic neurons of TPC DKO mice (red line, n = 57 cells, n = 14 mice) compared to control WT hypothalamic neurons (Ctrl, black line, n = 86 cells, n = 15 mice),  area under the curve (AUC). Statistical analysis were performed with two-way ANOVA followed by Sidak’s test. Values are expressed as mean ± SEM. *P < 0.05; **P < 0.01; ***P < 0.001.

In parallel with our neuropeptide release studies, we performed calcium imaging experiments in neurons from the hypothalamus to investigate whether TPCs are critical components of agonist-evoked Ca^2+^ responses underlying the neuropeptide secretion. Neurons from acute hypothalamic slices were loaded with the Ca^2+^-sensitive probe Calbryte 520-AM (Fig. 3*A*), and we recorded the global Ca^2+^ responses in both parvocellular and magnocellular neurons of the PVN. Neurons were first stimulated with either glutamate (Fig. 3 *B*–*E*) or DHPG (Fig. 3 *F*–*I*) to evoke a control response that was then challenged with various inhibitors. We analyzed three parameters: i) the Ca^2+^ elevation by measuring the increase in fluorescence ΔF/F_0_ at the peak of the response (Fig. 3 *B* and *F*), ii) the temporal course of the Ca^2+^ response, and iii) the total Ca^2+^ response measured as the area under the curve (AUC) (Fig. 3 *C*–*E* and *G*–*I*).

In the presence of either Ned-19 or GPN, the maximum peak response was reduced (Fig. 3 *B* and *F*), and the average Ca^2+^ responses evoked by either glutamate or DHPG showed important changes that consisted of a reduction in both the peak amplitude and the AUC (Fig. 3 *C*, *D*, *G*, and *H*). In TPC DKO mice, the time-course of the Ca^2+^ responses and the AUC were also measured upon glutamate (Fig. 3*E*) and DHPG (Fig. 3*I*) stimulation. When compared with WT mice, the responses were significantly reduced in TPC DKO mice, although in a less dramatic manner than with pharmacological inhibitors. However, these relatively modest reductions in the global Ca^2+^ responses in TPC DKO mice were associated with dramatic reductions in OT secretion (Fig. 2). These results are consistent with a critical role for endolysosomes and TPCs as a source of Ca^2+^, rather than nonspecific global intracellular Ca^2+^ signals (54), and highlight the importance of the Ca^2+^ source in neuropeptide secretion necessary for fine tuning the vesicular release mechanisms.

### Lysosomes and TPCs Control the Priming of OT Vesicles by Being in Close Apposition with LDCVs.

**Fig. 4.**
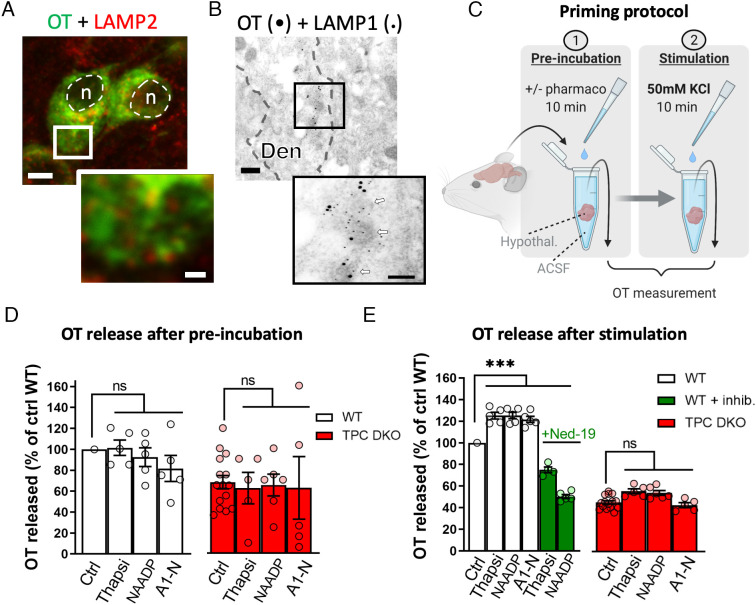
Lysosomes and TPCs control the priming of OT vesicles. (*A*) Confocal images in the PVN coimmunostained with OT (in green) and the lysosomal marker LAMP2 (in red). (Scale bars, 5 µm and 1 µm in the enlarged image.) (*B*) Electron micrograph of a dendrite (Den) from a SON neuron containing numerous neurosecretory OT vesicles (white arrows) identified by OT-associated neurophysin 1 immunoreactivity (15-nm gold particles). Lysosome distribution reveals by immunogold staining (6-nm gold particles) of LAMP1. Lysosomes appear surrounding the dense-cored vesicles and the membrane of electron lucent structures. (Scale bars, 200 nm, and 100 nm in the enlarged image.) (*C*) Scheme showing the priming protocol. (*D*) Basal levels of OT release measured in the supernatant after preincubation with 10 µM thapsigargin (Thapsi), 5 µM NAADP, or 10 µM of the TPC2 agonist TPC2-A1-N (A1-N) (Ctrl n = 10 mice; Thapsi n = 5 mice; NAADP n = 5 mice; A1-N n = 5 mice). Bars represent OT released in percentage of WT OT release without drugs. (*E*) OT secretion evoked by 50 mM KCl stimulation of the hypothalamus, following preincubation with various drugs. Bars represent OT released in percentage of WT OT release without drugs. Green bars show OT release in WT hypothalamus in response to thapsigargin (Thapsi) or NAADP preincubation in the presence of 100 µM Ned-19 (Ctrl WT and DKO n = 15 mice; thapsi + Ned-19 n = 4 mice; NAADP DKO n = 6 mice; all other groups n = 5 mice). Statistical analyses were performed with 1-way ANOVA followed by Dunnett’s test. Values are expressed as mean ± SEM. *P < 0.05; **P < 0.01; ***P < 0.001 and ns, nonsignificant.

Numerous studies in other cell systems have shown that secretion is highly dependent on the local Ca^2+^ from lysosomal stores (22, 55). Thus, we next addressed the question of whether lysosomes, the subcellular loci of TPCs, are strategically positioned to constitute a relevant intracellular Ca^2+^ store for neuropeptide secretion. We first examined lysosome distribution in neurons of PVN and SON by performing coimmunostaining of LAMP2, a lysosomal marker, with OT vesicles. Confocal images revealed that numerous lysosomes are situated in close proximity to OT-containing vesicles in the soma of magnocellular neurons both in WT mice and TPC DKO mice (Fig. 4*A* and *SI Appendix*, Fig. S4). Using electron microscopy, we also observed that in dendrites, lysosomes are in close apposition with OT vesicles (Fig. 4*B*), which agree with a local source of Ca^2+^ signal provided by TPCs activation to trigger OT release.

Small cytosolic Ca^2+^ concentration changes, dependent on Ca^2+^ release from internal stores, have been demonstrated to be important in the priming of nonreleasable vesicles into a readily releasable pool of vesicles, an essential process to trigger a sustained neuropeptide release (16, 56). In pioneering studies, Ludwig and Leng implicated the ER, the main intracellular Ca^2+^ store, in the mechanisms of priming (16). They used the SERCA pump inhibitor thapsigargin to release Ca^2+^ from the ER, which induced the priming of vesicles and therefore enhanced stimulus-induced neuropeptide secretion. We examined the role of lysosomal Ca^2+^ release in the priming mechanisms using a protocol based on the thapsigargin-induced Ca^2+^ release from the ER in isolated hypothalamus (16). In addition to thapsigargin, we also employed the TPC agonist NAADP and a new lipophilic synthetic membrane-permeant TPC2 agonist, TPC2-A1-N (a NAADP mimetic) (43). We preincubated hypothalamic tissues with these agents (Fig. 4*C*) and measured the release of OT before (Fig. 4*D*) and after KCl depolarization (Fig. 4*E*). In our experiments, thapsigargin alone did not stimulate neuropeptide secretion (Fig. 4*D*) but, as expected, after even a brief thapsigargin exposure for 10 min, the release of OT evoked by KCl was increased by 25% in WT mice compared with the control response (Fig. 4*E*). Similarly, incubation with either NAADP or TPC2-A1-N for 10 min did not trigger secretion by themselves (Fig. 4*D*) nor decreased hypothalamic OT content (*SI Appendix*, Fig. S5) but, strikingly, these TPC agonists primed KCl-induced OT secretion (+ 25% and + 21%, respectively) in WT mice (Fig. 4*E*). In addition, preincubation with the NAADP inhibitor Ned-19 prevented priming evoked by either thapsigargin or NAADP in WT mice (Fig. 4*E*). Furthermore, in TPC DKO mice, thapsigargin, NAADP or TPC2-A1-N treatments failed to prime the release from OT vesicles (Fig. 4*E*). Taken together, these data revealed that TPCs likely mediate the priming process of OT vesicles, which underlies their critical role in the control of neuropeptide release.

### Social Behavior Is Altered in TPC DKO Mice.

**Fig. 5.**
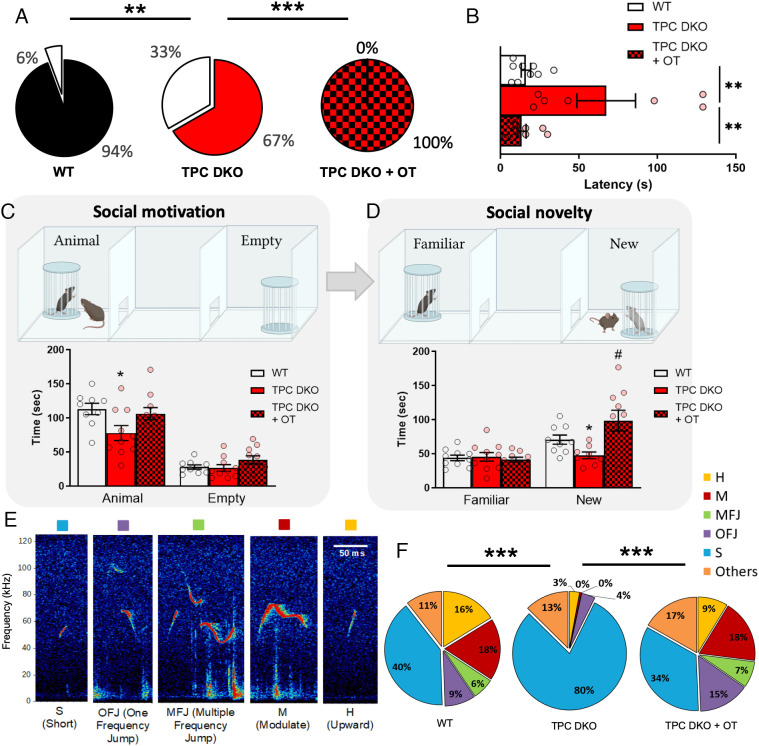
TPC deletion impaired maternal and social behaviors. (*A*) Proportion of pups retrieved by dams (WT n = 36, DKO n = 27, DKO+OT n = 30). (*B*) Latencies took by the dam to retrieve its first pup (WT n = 9, DKO n = 7, DKO+OT n = 10). (*C*) Diagram describing the social motivation test and histograms showing the time spent in contact with the empty pot or with the mouse visitor during the social motivation test. (*D*) Diagram describing the social novelty test and histogram showing the time spent with the familiar or the new mouse visitor during this test (WT n = 9 mice, DKO n = 10 mice, DKO+OT n = 10 mice). (*E*) Typical ultrasonic vocalizations (USVs) recorded during a free interaction between two adult males. (*F*) Proportion of each type of USVs emitted by the WT mice and the TPC DKO after intranasal NaCl administration (left and mid pie charts) and by TPC DKO mice after OT administration (right pie chart). Statistical analysis performed with *A* Fisher’s exact test, (*B*–*D*) unpaired two-tailed *t* test, (*F*) two-way ANOVA followed by a Chi-square post hoc test. Values are expressed as mean ± SEM. *P < 0.05; **P < 0.01; ***P < 0.001.

It is well known that the activity of OT neurons regulates social behavior and social interactions (for review see ref. 57). Because TPC DKO mice exhibit deficits in OT release (Fig. 2) and plasma OT levels (Fig. 1), we explored whether TPC DKO mice showed alterations in social behavior, both in females and males. To do so, we first analyzed the maternal behavior of TPC DKO dam mice. Although some controversy exists (58–60), most of the studies outline the prosocial effect of OT on maternal behavior (4, 5, 14, 21, 61–66). We performed a classical pup retrieval assay in which pups were placed at different corners of their home cage, and the dams’ task was to retrieve them and return them to the nest. During this task, the WT dams retrieved 94% of their pups with an average latency of 16 ± 3 s to retrieve the first pup (Fig. 5 *A* and *B*). In contrast, TPC DKO dams showed a reduced number of pups retrieved (67%) with a substantial increase in the average latency (67 ± 18 s) to retrieve the first pup (Fig. 5 *A* and *B*). To determine if the maternal deficit of TPC DKO mice could be restored, we administrated either a saline solution or OT (50 µg/kg) intranasally to TPC DKO dams. Strikingly, TPC DKO dams treated with OT now showed 100% success in retrieving their pups (Fig. 5*A*). The latency for retrieving the first pup also decreased significantly from 67 ± 18 s for NaCl-treated TPC DKO mice to 13 ± 2 s for OT-treated mice (Fig. 5*B*), a value comparable to that displayed by WT (16 ± 3 s).

**Fig. 6.**
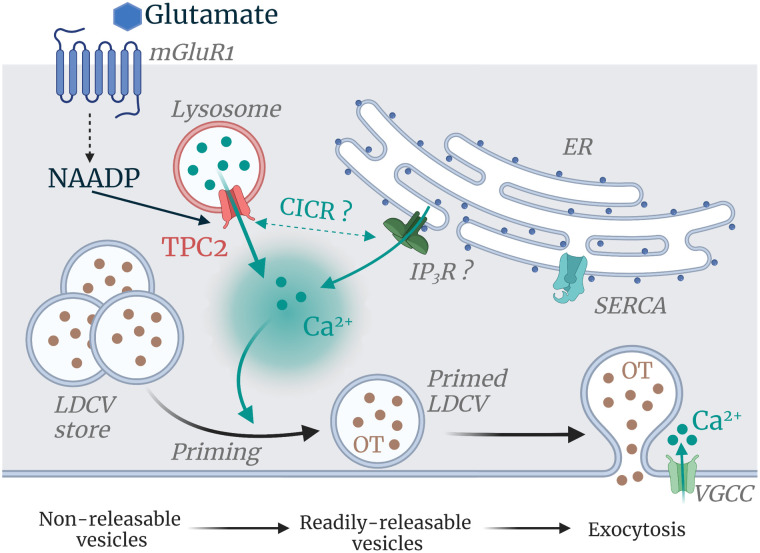
Endolysosomal TPCs control OT vesicle priming and release. Working model showing that glutamate, through mGluR1 activation, engages the NAADP pathway to stimulate Ca^2+^ release from endolysosomal TPCs. This Ca^2+^ response in its turns, participates in OT release likely by recruiting the ER Ca^2+^ by Ca^2+^-induced Ca^2+^ release. This scheme includes the priming working model we propose showing that TPCs generate the Ca^2+^ ionic signal to prime the nonreleasable vesicles into readily releasable vesicles either alone or by triggering a larger Ca^2+^ release from the ER stores (CICR). Note that for the priming process, the ER Ca^2+^ channels supporting priming remain to be identified. Abbreviations: mGluR, metabotropic glutamate receptor; CICR, Ca^2+^ -induced Ca^2+^ release; IP_3_R, IP_3_ receptors; LDCV, large dense core vesicle; SERCA, sarcoendoplasmic reticulum Ca^2+^ ATPase; VGCC, voltage-gated Ca^2+^ channel.

To determine whether TPC DKO adult male mice could also have altered social behaviors, we performed the 3-chamber test (Fig. 5 *C* and *D*) that enables the study of social motivation with a limited amount of social interaction and information (67) (no hierarchy, contact was limited to nose-pokes and olfactory information through holes). In the assay, TPC DKO mice spent significantly less time than the WT mice in exploring the unfamiliar congener (78 ± 10 s vs. 113 ± 8 s respectively, Fig. 5*C*). However, TPC DKO mice, like WT mice, spent significantly more time in social contact with the visitor mouse than with an empty cup (a nonsocial object, Fig. 5*C*). Next, the interest for social novelty was assessed by introducing a new visitor mouse in the empty cup. As expected, the WT mouse showed a significantly higher preference for interactions with the new congener than with the familiar mouse (Fig. 5*D*). In contrast, TPC DKO mice clearly failed to show any particular interest toward the new visitor (Fig. 5*D*). OT administration in TPC DKO mice restored both social motivation and the social novelty preference to levels seen in WT mice (Fig. 5*D*). Ultrasonic vocalizations (USVs), markers of emotional states associated with social context and OT signaling (62, 68, 69) (for review see ref. 70), were also recorded in a social environment allowing free interactions between adult male mice. Such a behavioral setting has been shown to favor the emission of acoustic communication in adult animals of same sex (71, 72). USVs emitted were counted, and no significant difference was observed between TPC DKO mice and WT mice (*SI Appendix*, Fig. S5). USVs were then further analyzed and sorted by types as illustrated in Fig. 5*E*. While WT mice exhibited a great diversity in their USVs repertoire, TPC DKO mice mainly emitted short vocalizations (Fig. 5*F*), shown to be used either by immature animals and/or by animals with low social incentives (71, 73). Strikingly, intranasal OT administration fully restored the USV proportion of the repertoire in TPC DKO mice, particularly USV with frequency modulation (Fig. 6*F*), a type of vocalizations that is not uttered in stressful conditions.

Additionally, to assess the specificity of the behavioral effects in TPC DKO mice, we also performed a test for which OT has not been implicated, namely fear conditioning. Fear learning and memory, assessed using a contextual fear conditioning paradigm, did not reveal any genotype differences during the acquisition session, or during recall/extinction of freezing responses in successive retention sessions (undertaken at 24 h, 48 h and 72 h delays) (*SI Appendix*, Fig. S6). Thus, TPC DKO mice do not have impaired associative learning ability when evaluated using a contextual fear conditioning test.

Finally, although the exact mechanism by which intranasal OT affects brain activity is debated (74, 75), our data indicate that representative social behaviors of both female and male TPC DKO mice are impaired and can be restored by OT administration. Thus, probably due to their role in OT release here, TPCs appear as new important regulators of social behaviors.

## Discussion

This study provides evidence of the important role of TPCs in the social brain as a molecular component of the LDCV priming response. Our work has revealed i) that deletion or pharmacological inhibition of TPCs reduces aspects of Ca^2+^ responses evoked by physiological agonists, which result in ii) a dramatic reduction in OT secretion from the hypothalamus and hypophysis. Furthermore, we showed that iii) TPC2 has a more prominent role than TPC1 in controlling OT secretion and that iv) TPCs participate in the priming process of neuropeptide vesicles. Finally, we showed that v) deletion of TPCs in mice leads to social defects that manifest in multiple domains of the animals’ social life such as maternal behavior, social motivation behaviors, and social communication and that all these defects can be rescued by intranasal OT administration.

Surprisingly, despite the numerous studies on OT, the regulation of its secretion is still poorly understood and represents a big gap in our understanding of OT signaling. Ca^2+^ influx through N- and T-type voltage-gated calcium channels and NMDA receptors participate in the OT release (30, 76), nevertheless, it has been previously shown that OT release mostly depends on intracellular Ca^2+^ stores (16, 56). Previous work focused on the ER as intracellular Ca^2+^ source. ER Ca^2+^ stores may be mobilized by inositol (1, 4, 5) trisphosphate (IP_3_) following G protein-coupled receptor activation (50), and by cADPR and other RyR modulators (21, 77). Through the synthesis of cADPR, the enzyme CD38 has been shown to be a major determinant involved in OT secretion (21, 77, 78). CD38 has also been previously linked to lysosomes and digestive enzyme secretion (25). In this study, we examined the other branch of the CD38 pathway, which catalyzes NAADP synthesis that mobilizes lysosomal Ca^2+^ stores through TPCs activation (for review (22, 42, 79). Our immunostaining and electron microscopy experiments revealed that lysosomes are strategically positioned Ca^2+^ stores since they are present at somatodendritic vesicular release sites. Next, by genetic or pharmacological ablation we have demonstrated that TPCs are major regulators of plasma OT levels and are required for the hypothalamic release of OT. Ultrastructural studies with electron microscopy provided additional evidence for the role of TPCs in secretion, since there was an accumulation of aged, nonreleased vesicles in neurohypophysial nerve endings of TPC DKO mice, consistent with a failure of their release. Since pharmacological or genetic ablation of TPCs reduces KCl-evoked somatodendritic OT release and there are reports describing the Ca2+-dependency of NAADP synthesis in various cell types (80–84). KCl-induced cell depolarization may promote NAADP production directly through Ca2+ influx mediated by voltage-gated Ca2+ channel activation. However, it is possible that the effect is indirect and a consequence of KCl-evoked transmitter release if that transmitter is coupled to NAADP production release, such as glutamate (85, 86). Given that there are extensive glutamatergic synapses with OT secreting neurons in the SON, we found that glutamate activation of mGluR1 evoked Ca^2+^ signals and OT secretion from dendrites, which was greatly reduced by Ned-19, a membrane-permeant selective inhibitor of NAADP-activation of TPCs, or when the lysosomal Ca^2+^ storage was abrogated by bafilomycin A1 (H^+^-V-ATPase inhibitor), or by GPN, a lysosomotropic agent, These pharmacological effects were all phenocopied in TPC DKO mice. These results are in agreement with a previous study in the hippocampus, where mGluR1 activation leads to the mobilization of intracellular Ca^2+^ through the production of NAADP and TPCs activation (31, 32). Despite the major reduction in OT secretion, highlighting the crucial role of TPCs as a major source for Ca^2+^ release in this process, mGluR1-induced Ca^2+^ signaling was not fully abolished with either a NAADP antagonist or in TPC DKO neurons. Indeed mGluRs are known to couple to additional Ca^2+^ mobilizing pathways such as cADPR or IP_3_, which release ER Ca^2+^ stores (22, 87–89). Together, our results show the specific involvement of TPCs in OT secretion, and we therefore propose a model for OT secretion (Fig. 6) whereby glutamate, by activating mGluR1, couples to the NAADP pathway, and evokes Ca^2+^ release from endolysosomes by activating TPCs. This source of local lysosomal Ca^2+^ release may in turn trigger further Ca^2+^ release from the ER by Ca^2+^-induced Ca^2+^ release (CICR) to participate in neuropeptide release. In this model, we also proposed that TPCs could mediate a CICR mechanism since TPC2 has also been shown to be a Ca^2+^-sensitive channel (22, 55). As NAADP levels in neurons have been shown to be elevated by glutamate stimulation (32), NAADP and Ca^2+^ may act as coagonists of TPCs.

Before being exocytosed, OT vesicles need to be recruited closer to the plasma membrane in a mechanism called priming. This essential and distinct feature permits magnocellular neurons to adapt neuropeptide release to the physiological needs and supports sustained neuropeptide secretion. Of note, within the hypothalamus, the OT secretion is principally somatodendritic, and it has been reported that nonreleasable vesicles are primed into readily releasable vesicles and triggered in an exclusive intracellular Ca^2+^-dependent manner (16). The downstream effects of the priming Ca^2+^ signals are not well understood. However, it has been proposed that the remodeling of the actin cytoskeleton may be involved (90). One source of Ca^2+^ for priming was identified as being the ER as hypothalamic incubation with thapsigargin enhance priming but with no role of RyR (16). The involvement of IP_3_R in priming also remains unclear, thus, the Ca^2+^ ER channel responsible of priming induction in the presence of thapsigargin remains to be identified. Here we showed that lysosomal Ca^2+^ release is a key mediated event involved in vesicle priming. Indeed, using a synthetic NAADP mimetic TPC2 agonist [TPC2-A1-N (43)] and NAADP, our results clearly indicate that Ca^2+^ mobilization through lysosomes and TPCs is important in priming as these agonists enhance OT secretion and failed to do so following pharmacological inhibition of TPCs or in TPC DKO mice. Additionally, our data showed that mobilizing ER Ca^2+^ stores to prime OT vesicle is also prevented by NAADP/TPC inhibition or deletion suggesting that a lysosomes-ER Ca^2+^ exchange is required for the priming effect (Fig. 6). This result is compatible with previous reports in pancreatic acinar cells and sea urchin eggs showing that Ca^2+^ release from the ER recruit the lysosomal Ca^2+^ demonstrating a bidirectional communication between ER and lysosomes (37, 81). Our model (Fig. 6) is supported by the recent reports showing that TPC2 is a Ca^2+^ sensitive channel that could be recruited by CICR mechanism (22, 55), which is in accordance with the close proximity we observed between lysosomes and OT vesicles in somatodendritic regions of the hypothalamus. Therefore, TPCs may provide the local Ca^2+^ signal needed for priming and in our model, TPCs, and especially TPC2, appear as a privileged pathway to trigger vesicle priming and thus increase subsequent OT secretion (Fig. 6).

In spite of the ubiquitous distribution of TPCs in the brain, study of the literature indicates that the role of TPCs in the central nervous system is largely unknown. Our study provides evidence that TPCs have important neurophysiological functions as they modulate the social brain by regulating neuropeptide secretion. In our study, we clearly showed that mice lacking TPCs displayed impaired maternal behavior, ultrasonic vocalization emissions and social recognition, which are social behaviors known to rely on several brain structures such as the prefrontal cortex, the hippocampus, the olfactory bulb, and the auditory cortex, all of which are reported to highly expressed OT receptors and to be modulated by OT release from axonal projection originating from PVN of the hypothalamus (5). In our study, we performed nasal-spray administration of OT, which has been successful in restoring normal social behaviors in TPC DKO mice. Although there is some debate over whether exogenous administrated OT could reach these brain structures (74, 75), numerous studies have shown that such OT administration protocols could be valuable to investigate OT effects on animal behavior (for review see refs. 15 and 91). Although we do not rule out that TPCs likely control many different neuronal processes, the simplest explanation for our behavioral data is to consider that TPCs are important, not only for somatodendritic or pituitary release of OT but also that TPCs can control the release of OT at nerve endings at distant sites from the hypothalamus, and in particular in the different brain structures involved in the social brain. In agreement with this possibility, it is striking that intranasal OT administration successfully restored the social behavior of TPC DKO mice, thereby revealing the peculiar importance of TPCs in the hypothalamic/social brain axis.

Our findings highlight the importance of lysosomes and a signaling pathway that has important implications for regulating social behavior. Finally, targeting TPC2 with agonists may offer a new therapeutic approach to potentiate the physiologically timed endogenous local release of OT in neurodevelopmental disorders characterized by social interaction deficits, thus bypassing current problems such as receptor desensitization with exogenous OT replacement therapies (92).

## Materials and Methods

### Animals.

*Tpcn1/2^−/−^* (TPC DKO) mice were generated as previously described (48). Mutants *Tpcn1^−/−^* (TPC1 T159) mice and *Tpcn2^−/−^* (TPC2 YHD437) mice used to generate TPC DKO are on a mixed genetic background of 129P2/OlaHsd and C57BL/6NCrl. Briefly, *Tpcn1*^+/−^/*Tpcn2*^+/−^ mice resulting from crosses of *Tpcn1^−/−^* (TPC1 T159) (93) with *Tpcn2^−/−^* (TPC2 YHD437) (26) mice were further crossed for generation of WT (*Tpcn1*^+/+^/*Tpcn2*^+/+^) and DKO mice (*Tpcn1^−/−^*/*Tpcn2^−/−^*) and mice were born at the expected Mendelian proportion (8/128) and kept as separate colonies bred in our animal facility. Consequently, in our study, the WT mice used as controls are not littermates of TPC DKO mice. Both mice strains have a mixed genetic background of 129P2/OlaHsd and C57BL/6NCrl and were not further backcrossed. Animal care and experimental procedures complied with the European Communities Council Directive (CEE 86/609/EEC), EU Directive 2017/32/EU, and the local ethics committee (Paris Centre et Sud, N°59).

### RNA Isolation, RT-PCR and Real-Time PCR.

Total RNA from pituitary and hypothalamus were extracted using Trizol reagent (Invitrogen). RNA concentrations were determined by NanoPhotometer^®^ N120 (IMPLEN). After reverse transcription using Super ScriptTM III Reverse Transcriptase (Invitrogen) from 1µg mRNA, 4 µL cDNA dilution (1:10) were added to 10µL iTaq Universal SYBR Green Supermix (BioRad, USA) and 0.5 mM of each specific pair primer.

Tpcn1 forward primer 5′CTGTCCTCTGGATGGAACCT3′;
Tpcn1 forward primer 5′CTGTCCTCTGGATGGAACCT3′;
Tpcn2 forward primer 5′CCCTGGCTGTATACCGATTG3′;
Tpcn2 reversed primer 5′GTCCCAGAGCGACAGTGG3′;
GAPDH forward primer 5′TGACGTGCCGCCTGGAGAAA3′;

GAPDH reversed primer 5′AGTGTAGCCCAAGATGCCCTTCAG3′ and then were amplified on a CFX96 Touch Real-Time detection system (BioRad) under the following conditions: 10-min denaturation step at 95 °C was followed by 40 cycles of denaturation at 94 °C for 10 s and annealing/extension at 60 °C for 30 s. The results were normalized to GAPDH, and relative mRNA levels were calculated using the ΔΔCt method and reported as fold change (relative to WT).

### Generation and Analysis of tGFP-TPC2 Mice.

*tGFP-TPC2 mice* were generated and analyzed as described in ref. 49 and in the *SI Appendix*. Briefly, the brain and hypophysis were frozen in Tissue-Tek O.C.T. compound (4583, Sakura), and 14-µm cryosections were prepared. The slices were incubated with a chicken-GFP antibody (Invitrogen, A10262), diluted at 1:1,000, overnight at 4 °C. After washing, the sections were incubated with donkey anti-chicken-Cy2 (Jackson Immunoresearch Cat. 703-225-155), diluted at 1:500, for 2 h at room temperature. Nuclei were stained for 5 min using 2 µg/mL bisbenzimide solution (Sigma, B1155) before mounting in Fluoromount-G (Biozol, SBA-0100-01). Images were acquired using a Zeiss AxioScan.Z1 slide scanner and processed using ZenBlue software.

### Blood Sampling.

Mouse blood samples were collected in a homemade EDTA tube by cardiac puncture under profound Exagon anesthesia (150 mg/kg) and then centrifuged to collect the plasma.

### Hypothalamus Sampling and Stimulation.

Whole-mouse hypothalami were rapidly and carefully removed from the brains and perifused with oxygenated artificial cerebrospinal fluid (ACSF) (see composition in the “*Calcium Imaging*” part, 5% CO_2_, 95% O_2_) for 30 min at 33 °C. Such perifusion aimed to remove the potential OT secreted due to mechanical stimulation of the hypothalamus during the dissection and prevent the auto-stimulation effect of OT on its receptors. During the preincubation step, hypothalami remained untouched while the perfused ACSF was removed. Hypothalami were then immediately bathed in 200 µL ACSF for 10 min. When no drugs are added, the collection of the supernatant aimed to measure the basal release of OT. Depending on the experiment, the preincubation ACSF solution contains drugs: DMSO (vehicle), Ned-19 (100 µM), tetrandrine (10 µM), bafilomycin A1 (4 µM), and GPN (50 µM). The second step aimed to stimulate OT release. Hypothalami were carefully bathed for 10 min in 200 µL ACSF containing either KCl (50 mM), glutamate (1 mM), or DHPG (100 µM). Here, the collection of the supernatant aimed to measure the stimulated release of OT. At the end of the experiments, the hypothalami and the supernatant solutions (basal release and stimulated release) were collected and frozen in liquid nitrogen.

### Priming Experiment.

Sampling and perifusion of the hypothalamus were similar to those described in “*Hypothalamus Sampling and Stimulation*”. Here, the first step was stimulation of the vesicle priming with ACSF containing either thapsigargin (10 µm), NAADP (5 µM), TPC2 agonist TPC2-A1-N (A1-N, 10 µM), or classic ACSF for the control unprimed samples. The agonist TPC2-A1-N was kindly synthesized and provided by Franz Bracher’s lab (43). If priming occurs, more vesicles would be close to the plasma membrane and available for secretion during a subsequent stimulation. Thus, during a second step, OT secretion was stimulated with high KCl (50 mM) in the absence of any pharmacological agent, and the amount of neuropeptide released was compared with the unprimed condition. The protocol is depicted in Fig. 4*C*.

### Radioimmunoassays for OT Measurements.

The extraction for OT was performed at 4 °C and measured as fully described in Martucci et al., 2019 (78) and in the *SI Appendix*.

### Immunohistochemistry.

Brains were removed after perfusion of the animals with paraformaldehyde PFA 4% and then frozen and stored at −20 °C until immunochemistry. Forty-µm-thick hypothalamic slices were obtained with a cryostat slicer and immediately washed with a phosphate buffered saline (PBS) solution (in mM: 35,4 NaH_2_PO_4_*2H_2_O, 160.7 Na_2_HPO_4_*2 H_2_O, 154 NaCl). Slices were then unmasked with citrate pH6 in the microwave before being first washed with a PBS solution. Slices were then washed twice with PBST (1L PBS + 2 mL Triton). A quenching solution composed of PBS + NH_4_Cl 50 mM was then used to prevent autofluorescence of the remaining PFA. Slices were washed with PBST; and treated with Smart 1% and normal goat serum (NGS 5%, Abcam ab7481) before being incubated in primary antibodies (Rat monoclonal IgG anti-LAMP2, Abcam ab13524, 1/1,000; Rabbit polyclonal anti-OT, Peninsula Lab T4084, 1/500) in Smart (1%) + NGS (5%) in PBST at 4 °C overnight. After the primary antibodies’ incubation, slices were washed in PBST before being incubated with secondary antibodies (Goat polyclonal anti-rat 568, Abcam ab175476, 1/400, and Goat polyclonal anti-rabbit 488, Abcam ab150081, 1/600) for 2 h. Slices were then first washed twice with PBS and then with PB (in mM: 35.4 NaH_2_PO_4_*2H_2_O, 160.7 Na_2_HPO_4_*2 H_2_O) before being coverslipped with fluoromount DAPI (Abcam ab104139). Slices were observed under a Nikon eclipse FN1-A1R confocal microscope (×40) and analyzed with the Software NIS Element AR4.50.00. Images were acquired by sequential scanning.

### Electron Microscopy.

Brains and pituitaries were removed after perfusion of the animals with PFA (4%) + glutaraldehyde (0.1%). The preparations were incubated with primary antibodies (Rat monoclonal IgG anti-LAMP1, mouse monoclonal anti-OT, Millipore, 4G11, mouse monoclonal IgG anti-OT-Neurophysin I) (94) for 1 h. After incubation with the primary antibodies, the sections were incubated with a goat antibody against rabbit IgG or mouse IgG conjugated to gold particles (5, 10, or 15 nm) (BBI Solutions) for 1 h. Sections were first incubated with the biotinylated goat anti-rat IgG for 10 min, followed by incubation in avidin-biotin-horseradish peroxidase (HRP) complex solution for 5 min. The sections were then washed with PBS, incubated with the goat antibody against HRP conjugated to 6-nm gold particles (Jackson ImmunoResearch Laboratory) for 1 h. Finally, the sections were contrasted with uranyl acetate and lead citrate and viewed using an H-7650 (Hitachi) electron microscope operated at 80 kV. For details see *SI Appendix*.

### Calcium Imaging.

Coronal acute slices (250-µm thickness) of the hypothalamus were obtained from 2 to 3-mo-old male mice. They were cut using a vibratome (Microm France) in a low Ca^2+^ ACSF containing (in mM): NaCl, 142; Hepes, 10; glucose, 10; CaCl_2_, 1; KCl, 1.5; KH_2_PO_4_, 1.25; MgCl_2_, 1.5 (pH 7.4, 310 to 330 mOsm) oxygenated at 4 °C. Slices were then incubated for 1 h at 35 °C in a classic ACSF containing (in mM): NaCl, 126; Hepes, 10; glucose, 10; CaCl_2_, 2; KCl, 1.5; KH_2_PO_4_, 1.25; MgCl_2_, 1.5 (pH 7.4, 310 to 330 mOsm) and oxygenated continuously with a mixture of 95% O_2_ and 5% CO_2_. Slices were loaded with the calcium probe (Calbryte 520 AM diluted in Pluronic acid) at 10 µM for 1 h at 33 °C in an ACSF solution oxygenated continuously with a mixture of 95% O_2_ and 5% CO_2_. After being incubated, slices were observed under a Nikon eclipse FN1-A1R confocal microscope and analyzed with the Software NIS Element AR4.50.00.

### Pups’ Retrieval Test.

The home cage (25 × 15 × 13 cm) is used in the experiment where three pups (P3 to P4) of the litter are placed in each opposite corner of the nest. The experiment began when the dam is placed on her nest. Time is manually measured to determine how long the mother takes to bring their pups back to the nest. The experiment ends after 3 min, even if no pups or not all the pups are retrieved. The number of pups retrieved is also evaluated.

### Three-Chamber Social Behavior Test.

Tested male mice were isolated 1 wk before the experiment. Mice were aged between 4 and 7 mo. The three-chambered apparatus was under 100 lux illumination, constituted of transparent Plexiglas, and contains three compartments (Fig. 5 *C* and *D*). The TPC DKO or WT mouse tested could freely explore the environment for 10 min before the experiment starts. The experiment was then divided into two successive tasks delayed by 5 min: the social motivation trial and the novelty motivation trial. During the social motivation trial, an unknown WT mouse was placed under a cup (the social cup) in one of the compartments while an empty cup (or nonsocial cup) was placed in the opposite compartment. The tested mouse was then placed in the central compartment of the apparatus and could freely explore the apparatus for 10 min. In the second trial, a new WT mouse was placed under the empty cup while the previous WT mouse used during the first trial remained under its cup (the “familiar mouse”). The tested mouse placed in the middle compartment could freely explore the apparatus for 10 min. During all experiments, the number and duration of contacts were scored as well as exploration reflected by the scoring of rearing events using video recording and analyzed with the Any-maze software (Stoelting, USA).

### Ultrasonic Vocalization Recordings.

Adult male mice previously isolated for 3 wk were left alone to explore a novel arena (50 cm × 25 cm) containing clean bedding for 30 min before an unknown conspecific -same age, sex, and WT-genotype was gently introduced in the arena for a 8-min free social interaction during which USVs were recorded (72). As previously described in detail elsewhere (71), an ultrasound microphone placed above the arena allows the continuous recording of USVs while mice are having free and reciprocal social interactions. The recording and analyses were performed with Avisoft Recorder and SASLab Pro software from Avisoft Bioacoustics (Berlin, Germany). USVs were classified into 10 categories following the shape of the spectrograms (Fig. 5*E*) as well as the duration and frequency modulations of the calls.

### OT Administration.

The amount and concentration of OT delivered intranasally varies very considerably in the literature, from 9.6 µg/kg (95, 96) to 666 µg/kg in rodents (97, 98). and we chose an intermediate concentration of 50 µg/kg. Prior to behavior tests, OT (Tocris Bioscience) or NaCl for controls, was administered intranasally under rapid isoflurane anesthesia (5%, 0.8 mL/min O_2_ for 2 min) to allow deep and regular breathing. The animal was then put back into its home cage for at least 30 min before the experiment.

### Statistical Analysis.

Proportions were compared using the Chi squared test. Normality was evaluated with the Shapiro–Wilk test. When data did not follow a normal distribution, we used the nonparametric Mann–Whitney two-tailed test for independent comparisons and the signed-rank Wilcoxon test when data were paired. When data followed a normal distribution, we used the Student two-tailed *t* test. For multiple comparisons, 1-way or 2-way ANOVAs were used followed by post hoc test. Multiple comparisons of nonparametric samples were performed using a Kruskal–Wallis test. Each value is expressed as mean ± SEM, and significances were illustrated with the following conventions: ns *P* > 0.05, *P < 0.05, **P < 0.01, ***P < 0.001.

## Supplementary Material

Appendix 01 (PDF)

## Data Availability

All study data are included in the article and/or *SI Appendix*.
