# Supplementary material for: Endolysosomal TPCs regulate social behavior by controlling oxytocin secretion
Source: Proc Natl Acad Sci U S A. 2023 Feb 6;120(7):e2213682120. doi: 10.1073/pnas.2213682120 (PMC9963339; doi:10.1073/pnas.2213682120)
Supplement: Supplementary file 1 — Appendix 01 (PDF) [file pnas.2213682120.sapp.pdf]

PNAS

## Supporting Information for

# Endolysosomal two-pore channels (TPC) regulate social behaviour by controlling oxytocin secretion

Lora L Martucci[1,4,9*], Jean-Marie Launay[2], Natsuko Kawakami[3], Cécile Sicard[1], Nathalie Desvignes[1], Mbarka Dakouane-Giudicelli[4], Barbara Spix[5], Maude Têtu[1], Franck-Olivier Gilmaire[1], Sloane Paulcan[1], Jacques Callebert[6], Cyrille Vaillend[1], Franz Bracher[7], Christian Grimm[5], Philippe Fossier[1], Sabine de la Porte[4], Hirotaka Sakamoto[3], John Morris[8], Antony Galione[9*], Sylvie Granon[1], José-Manuel Cancela[1*]

*Correspondence: lora.martucci@new.ox.ac.uk ; antony.galione@pharm.ox.ac.uk ; jose-manuel.cancela@universite-paris-saclay.fr

**This PDF file includes:**
Supplementary methods and Figures S1 to S6

## Generation and analysis of *tGFP-TPC2 mice*

*tGFP-TPC2 mice* were generated and analyzed as described in (49). Briefly, neonatal mice (6 days of age) were transcardially perfused with PBS, followed by PFA (4%). The brain and hypophysis were removed and subjected to a post fixation in PFA (4%) for 2-4 hours, before incubation in sucrose solution (18%) overnight for cryoprotection. Then, the brain and hypophysis were frozen in Tissue-Tek O.C.T. compound (4583, Sakura) and 14 µm cryosections were prepared. Tissue sections were blocked for 1 hour at room temperature in a solution of PBS (1X) containing normal donkey serum (10%, Jackson Immunoresearch Cat. 017-000-121), BSA (3%) and Triton-X-100 (0,3%). Then, slices were incubated with a chicken-GFP antibody (Invitrogen, A10262), diluted at 1:1000, overnight at 4°C. After washing 3 x 5 min in PBS (1X) containing Tween-20 (0,05%), the sections were incubated with donkey anti-chicken-Cy2 (Jackson Immunoresearch Cat. 703-225-155), diluted at 1:500, for 2 hours at room temperature. Nuclei were stained for 5 min using 2 µg/ml Bisbenzimide solution (Sigma, B1155) before mounting in Fluoromount-G (Biozol, SBA-0100-01). Images were acquired using a Zeiss AxioScan.Z1 slide scanner and processed using ZenBlue software.

## Radioimmunoassays for OT measurements

The extraction for OT was performed at 4°C as fully described in Martucci et al., 2019 (78). After extraction, the OT content was estimated using a highly sensitive and specific radioimmunoassay (RIAgnosis, Munich). In brief, 0.05 mL of the rabbit anti-OT antibody was applied to the extract for 60 min. Next, 0.01 mL of $^{125}$I-labeled tracer (Perkin Elmer) was added to each aliquot. After an incubation period of 3 days at 4°C, unbound radioactivity was precipitated by activated charcoal (Sigma Aldrich).

## Electron microscopy

Brains and pituitaries were removed after perfusion of the animals with PFA (4%) + glutaraldehyde (0.1%). Post-fixation in PFA (4%) overnight, then washed in PBS before electron microscopy. The preparations were dehydrated through increasing concentrations of methanol and embedded in LR Gold resin (Electron Microscopy Sciences). Ultrathin sections were then incubated with normal goat serum (2%) and BSA (2%) in 50 mM tris(hydroxymethyl)-aminomethane buffered saline (TBS; pH 8.2) for 30 min to block non-specific binding. The sections were then incubated with primary antibodies (Rat monoclonal IgG anti-LAMP1, mouse monoclonal anti-OT, Millipore, 4G11, mouse monoclonal IgG anti-OT-Neurophysin I (93) for 1 h. After incubation with the primary antibodies, the sections were washed with PBS, then incubated with a goat antibody against rabbit IgG or mouse IgG conjugated to gold particles (5, 10 or 15 nm) (BBI Solutions) for 1 h. To intensify the detectability of the immunoreaction for LAMP1, a streptavidin-biotin intensification kit (Nichirei) was used. Sections were first incubated with the biotinylated goat anti-

rat IgG for 10 min, followed by incubation in avidin-biotin-horseradish peroxidase (HRP) complex solution for 5 min. The sections were then washed with PBS, incubated with the goat antibody against HRP conjugated to 6 nm gold particles (Jackson ImmunoResearch Laboratory) for 1 h. Finally, the sections were contrasted with uranyl acetate and lead citrate and viewed using an H-7650 (Hitachi) electron microscope operated at 80 kV. The number of endings containing autophagic vacuoles in the posterior pituitary was analyzed among WT, TPC DKO, and Tpcn2-/- group. We analyzed, at ×3,000 magnification, montage photographs by 25 images (unit area: 30 × 30 μm2) in the posterior pituitary containing OT-positive endings. At least, 6 montage photographs were obtained for each mouse. In total, 18-unit areas were analyzed. We scored the autophagic vesicle status as following: the grade 0 corresponds to an absence of autophagic vacuoles within endings in the posterior pituitary, whereas grades 1, 2, and 3 correspond to a progressive increase in the number of autophagic vacuoles: grade 1 (one or more autophagic vacuoles were observed), grade 2 (autophagic vacuoles accounted for more than half), and grade 3 (autophagic vacuoles accounted for more than 80%). The number of endings in each genetic group was analyzed. Statistical analyses were performed using two-way analysis of variance (ANOVA). When significant main effects were found using ANOVA, the post hoc Tukey-Kramer test was performed. The area of endings containing autophagic vacuoles in the posterior pituitary was analyzed among WT, TPC DKO, and Tpcn2-/- group. We analyzed, at ×3,000 magnification, montage photographs by 9 images (unit area: 18 × 18 $\mu m^2$) in the posterior pituitary containing OT-positive endings. At least, 6 montage photographs were used for each mouse. In total, 18-unit areas were analyzed. Three autophagic categories (grade 1–3) were also used as described above. The area of endings in each genetic group was analyzed. Statistical analyses were performed using Williams test. All the various analyses in this study were conducted ‘blind’.

**Contextual fear conditioning**

The conditioning box consisted of a grid floor (30 x 3 x 30 cm) enabling delivery of electric foot shocks as unconditioned stimuli (UCS) and clear Plexiglas walls (45 cm in height) with no ceiling in order to allow full observation and video tracking. Experiments were performed under moderate illumination (150 lux). During acquisition (day 1, duration: 8 min), each mouse was allowed to freely explore the box for 2min; 3 foot shocks (0.4mA, 2 s) were then successively delivered with a 2-min interval between shocks (2, 4, 6min), and the mouse remained in the box for 2min after the last shock. Retention and extinction of conditioned fear was measured during the next 3 days (24, 48, 72 h) by placing the mouse in the same context for 20 min during each daily session without any foot shock. Tonic immobility (freezing) was analyzed from video-tracking plots (AnyMaze) and used to quantify expression of fear responses and fear memory.

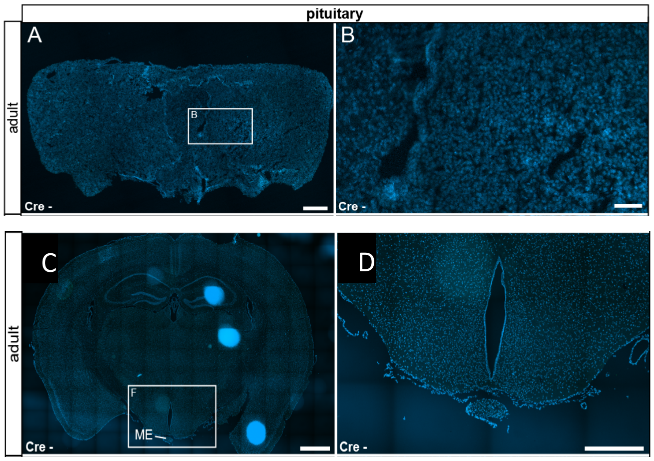


Supplemental figure S1: Immunostaining control experiments with the antibody anti-GFP (Chicken anti-GFP #A10262, Invitrogen) in Cre negative (-) control mice of the TPC2-tGFP reporter mice and nuclei stained with bisbenzimide (in blue) in the hypothalamus. Top upper row shows the pituitary gland with zoom-in image on the right site. The bottom row shows the PVN, SON and third ventricle (3V) with zoom-in image of the PVN/SON region on the right site. Top row scale bar = 200 µm and 50 µm for the zoom-in image. Bottom row scale bar = 1000 µm and 500 µm for the zoom-in image.

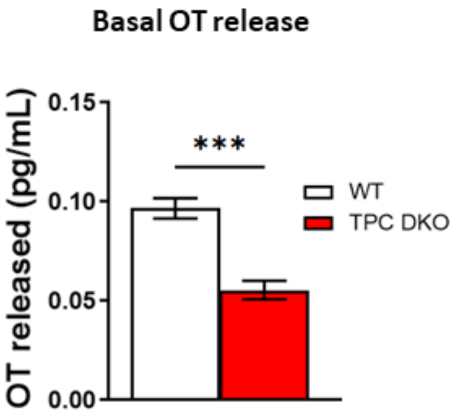


**Fig. S2.** Histogram showing the basal levels of OT released from unstimulated isolated hypothalami (WT n=24 mice; DKO n=20 mice). Statistical analysis were performed by the two-tailed t-test.

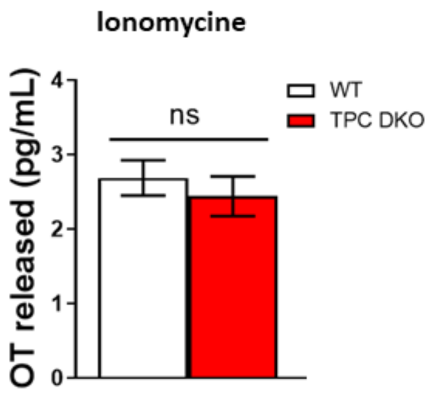


**Fig. S3.** Histogram showing measurement of OT released from isolated hypothalami upon ionomycin stimulation (WT n=5 mice; DKO n=5 mice). Data are expressed as mean ± s.e.m.. Unpaired t-test ***p<0.001

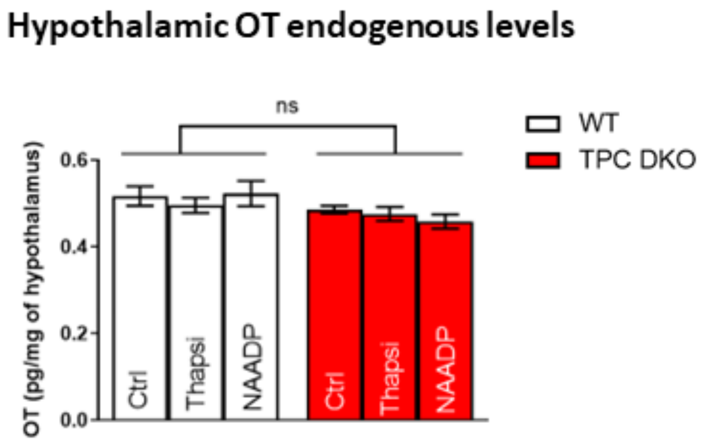


**Fig. S4.** Histogram showing the endogenous tissue content of OT measured in isolated hypothalami at the end of the priming experiments in presence of 10 µM thapsigargin (Thapsi) or 5µM NAADP. Data are expressed as mean ± s.e.m. Unpaired t-test.

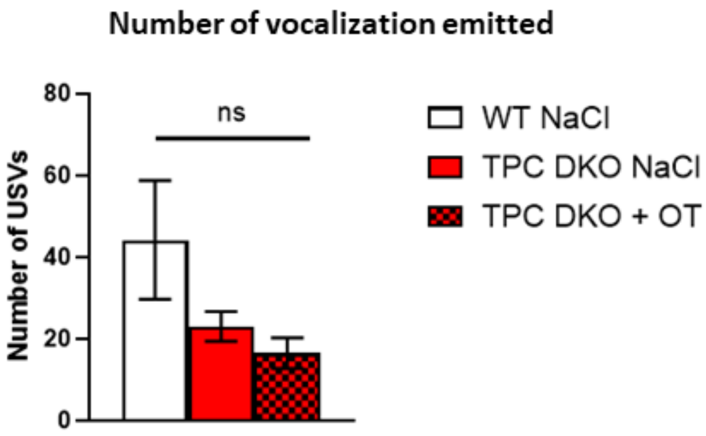


**Fig. S5.** Histogram showing the number of ultrasonic vocalizations (USVs) recorded during a free social interaction between adult male mice after intranasal administration of NaCl or OT (WT n=6 mice ; DKO n=9 mice ; DKO + OT n=9 mice). Data are expressed as mean ± s.e.m.. ANOVA 2-ways followed by a Fischer post-hoc test.

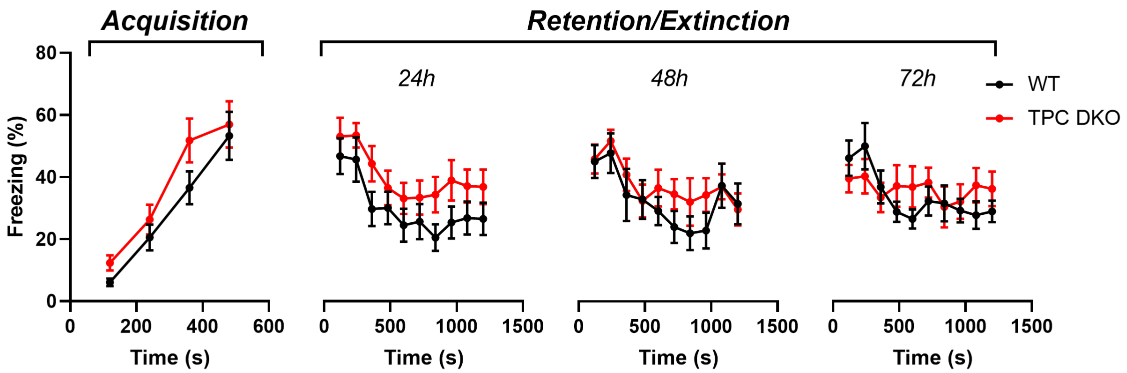


**Fig. S6**. Contextual fear conditioning. The plot shows the percent time spent freezing recorded in WT (n = 10 mice) and TPC-KO mice (n =9 mice) per time bins of 120s, during the acquisition session (8 min, 3 shocks delivered at 2, 4 and 6 min) and during successive retention sessions (24, 48, and 72 h) that enabled to quantify memory retention performance at recall (first point in each daily curve) and extinction of the freezing response during the daily 20-min recording period. No significant genotype difference was detected in all sessions (2-way ANOVAs).
